# An Elliptic Curve Based Schnorr Cloud Security Model in Distributed Environment

**DOI:** 10.1155/2016/4913015

**Published:** 2016-02-15

**Authors:** Vinothkumar Muthurajan, Balaji Narayanasamy

**Affiliations:** ^1^Department of CSE, University College of Engineering, Dindigul, Tamil Nadu 624622, India; ^2^Department of Information Technology, KLN College of Engineering, Madurai, Tamil Nadu 630612, India

## Abstract

Cloud computing requires the security upgrade in data transmission approaches. In general, key-based encryption/decryption (symmetric and asymmetric) mechanisms ensure the secure data transfer between the devices. The symmetric key mechanisms (pseudorandom function) provide minimum protection level compared to asymmetric key (RSA, AES, and ECC) schemes. The presence of expired content and the irrelevant resources cause unauthorized data access adversely. This paper investigates how the integrity and secure data transfer are improved based on the Elliptic Curve based Schnorr scheme. This paper proposes a virtual machine based cloud model with Hybrid Cloud Security Algorithm (HCSA) to remove the expired content. The HCSA-based auditing improves the malicious activity prediction during the data transfer. The duplication in the cloud server degrades the performance of EC-Schnorr based encryption schemes. This paper utilizes the blooming filter concept to avoid the cloud server duplication. The combination of EC-Schnorr and blooming filter efficiently improves the security performance. The comparative analysis between proposed HCSA and the existing Distributed Hash Table (DHT) regarding execution time, computational overhead, and auditing time with auditing requests and servers confirms the effectiveness of HCSA in the cloud security model creation.

## 1. Introduction

**Figure 1:**
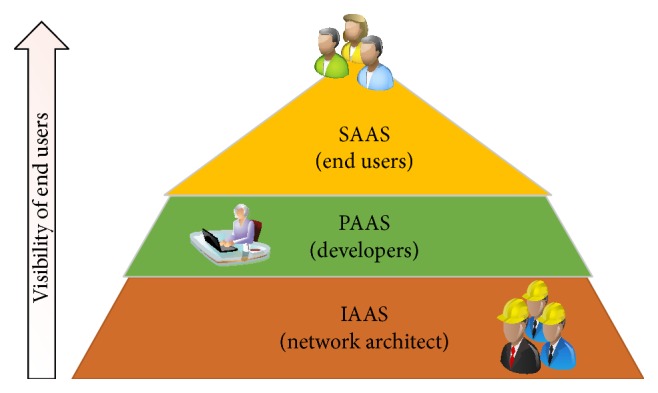
Cloud services.

Cloud computing intensifies the Information Technology (IT) architecture with the following advantages: on-demand self-service, resource elasticity, and shared pool access. The objective of cloud paradigm is to share the data computations over the scalable network nodes, namely, user computers, cloud services, and data centers. Several grades of services are available in the cloud architecture, namely, Software As A Service (SAAS), Platform As A Service (PAAS), and Infrastructure As A Service (IAAS) as shown in Figure 1. IAAS describes the consumer ability to handle the provisional processing by using conventional resources. PAAS denotes the deployment of consumer-created applications into the cloud structure. SAAS defines the running process of provider's applications on the cloud structure. The movement of data to cloud raised the integrity challenges in the auditing process. The cloud services auditing assures the remote data integrity.

The higher data burden consumes more time. Hence, new methods are required to reduce the burden of metadata in the cloud services for better security. The data storage auditing system carries three modules, namely, owner, auditor (Third Party Auditor (TPA)), and server. The TPA audits the owners and servers in the system model. Several privacy-preserving, dynamic audit service protocols govern the auditing process. The cloud services relying on the network infrastructures suffer the various attacks such as replace, forging and reply attacks that affect the security. Key management schemes such as symmetric (compact key, pseudorandom functions) and asymmetric algorithms (RSA, DES, AES, and Blowfish) are applied to a cloud computing model to ensure the security.

The key length of the cryptographic mechanism should be maintained for higher security that leads to more computational workload. The Elliptic Curve Cryptography (ECC) is applied to cloud service model to overcome the problems of computational load and key length maintenance. The optimization of servers and the allocation of data centers are achieved by a virtual machine (VM) based cloud computing. The overloading effect of server moves the VM into an energy model in which temperature based resource utilization is performed to analyze the degree of overloading effect. But the secure data transfer is an investigating process in an energy model.

The blooming filter process in cloud computing improves the data security. The system consists of three modules, namely, Aggregation and Distribution (AD), users, and clouds. The utilization of multiple ADs reduced the communication cost. But the retrieval of matched files further improves the reduction of cost in demand. This paper addresses the security problems in cloud computing and discusses the solution by ECC. Initially, a reconfiguration of the traditional ECC model with the new key generation mechanism to improve the security and reduce the execution time is carried out. Then, blooming filter concept application in proposed ECC-based model removes the irrelevant resources from server to improve the auditing efficiency and malicious activity prediction. The proposed blooming filter-ECC-based model analyzes the performance parameters of execution time, storage complexity, and security which confirms the effectiveness.

This paper is organized as follows: Section 2 describes the related works on cloud security model and auditing process.  Section 3 discusses the proposed Hybrid Cloud Security Algorithm (HCSA) implementation.  Section 4 presents the performance analysis of HCSA regarding security, execution time, and storage complexity. Finally, Section 5 presents the conclusion.

## 2. Related Work

This section discusses the traditional research works on the auditing process and addressed the security issues in the cloud service models. Wang et al. submitted the auditable cloud data storage to validate the data hosting on the network architecture [1]. The secure data hosting to the cloud was affected by the different identities of data owners and servers. Yang and Jia proposed an independent auditing service for host monitoring. They suggested the auditing protocol requirements and analyzed the existing auditing process on the security and performance [2]. The growth of cloud computing model depends upon the security challenges. Kulkarni et al. introduced the detailed analysis of security challenges in cloud computing system and the service delivery types [3]. The outsourcing through the untrusted cloud leads to insecure model. Zhu et al. constructed the dynamic audit service based on the index hash table and fragment structure techniques [4]. The performance of services was improved by a probabilistic query and periodic verification. The privacy vulnerabilities and online burden in fragmentation lead to the security problem. Wang et al. proposed the secure cloud storage system based on Privacy-Preserving Public Auditing (PPPA) [5] to reduce the vulnerabilities. They extended the results to offer simultaneous multiusers auditing. Yang and Jia designed an auditing framework for efficient cloud storage systems based on PPPA [6] for dynamic operations of data. The PPPA based cloud computing effectively reduced the computation cost of the audit. The auditing services address the risk issues in data access.

Li et al. performed Attributes-Based Encryption (ABE) schemes on Personal Health Record (PHR) [7] file. They focused cloud computing model on three aspects, namely, multiple data owner scenarios, a division of the multiple users into security domain, and complexity reduction in key management policies. The encrypted data suffered from the multikeyword search problem in multisecurity domain. Cao et al. solved the challenging problem of Multikeyword Ranked Search Encryption (MRSE) [8] that improved the privacy requirements. Ryan briefly analyzed the issues related to secure cloud computing model creation. The data sharing between the service providers based on symmetric key management schemes is regarded as a core scientific problem [9]. The data sharing in cloud storage depended on the factors such as security and flexibility. Chu et al. presented the aggregate cryptosystem [10] for data sharing. The aggregate key released by key holder according to flexible choice and the other keys were kept confidential.

Hwang et al. separated the encryption/decryption mechanisms by using the Customer Relationship Management (CRM) [11] service. CRM provided suggestions for multiparty Service Level Agreement (SLA). The security requirements were specified by using CRM according to privacy issues. Suo et al. discussed the processing groups of cloud service model encryption, communication security, and protection of sensor data [12]. Jager et al. extended the cloud service model by considering the unrestricted attacks [13] to sealed cloud. The data confidentiality was poor in sealed cloud, which was improved by new Cipher cloud. Kaur and Singh ensured the data confidentiality by two encryption schemes [14]. The data transfer between cloud server and client is encrypted and kept confidential in cipher cloud.

Key Distribution Centers (KDC) distributed keys to both the users and the cloud servers where a single key was replaced by the multiple keys of the owners. Ruj et al. proposed the Distributed Access Control in Cloud (DACC) algorithm [15] for KDC. They applied Attribute-Based Encryption (ABE) by a pairing of elliptic curves. The unique KDC-based cloud computing inherited the security issues. The insolubility of mathematical problems in KDC dealt with the new cryptography scheme termed as Elliptic Curve Cryptography (ECC). Chakraborty et al. presented homomorphic encryption scheme [16] based on ECC. Fast data access was performed by using the Merkle Hash Tree (MHT) at the server. The data leakage problem was not considered in a homomorphic scheme. Lee and Chen presented the cloud aided computation with elliptic curve cryptosystems [17] to deal with the leakage problem. They also prevented the active and passive attacks such as guessing and modification attacks. Cloud Service Providers (CSP) required updating and scaling of data on remote servers. Barsoum and Hasan performed the data outsourcing from owner to CSP with mutual trust between the CSPs by using outsourcing algorithm [18]. Chen et al. proposed secure outsourcing algorithm [19] to untrusted program models and achieved the secure encryptions and signatures. The energy consumption was more in traditional cloud computing processes.

Goudarzi and Pedram utilized the virtual machine [20] and server consolidation in the data center to reduce the energy consumption. The resource requirement reduced significantly by using virtual machine model. The dynamic allocation of data centers was an important et al. presented the virtualization based system for optimization [21]. They also introduced the concept of skewness to measure the dissimilar items in the multidimensional resource utilization. The deployment of virtual machine required computing resources. Shiraz et al. analyzed the effect of virtual machine deployment [22] at the execution time. The migration cost of virtual machine altered in accordance with configurations and workloads. Liu et al. predicted the performance of migration and the cost of energy quantitatively by hypervisor virtual machine model [23]. The hypervisor virtual machine model was evaluated on representative workloads. The usage of resources was efficiently enhanced by deduplication technique. But deduplication suffered from security weakness. Blasco et al. presented the solution based on bloom filter [24] for efficient deduplication. They provided the description about bloom filter and compared the solution through security analysis by using extensive benchmarking sets. The search time of text in encrypted documents was more. Pal et al. reported the novel approach for storing the data in a remote server and the searching process in constant time without degradation [25]. The cloud security model analyzed by an enhanced bloom filter with the EC-Schnorr based encryption scheme is presented in this paper.

## 3. Elliptic Curve Based Schnorr Model for Cloud Security Improvement Using HCSA 

**Figure 2:**
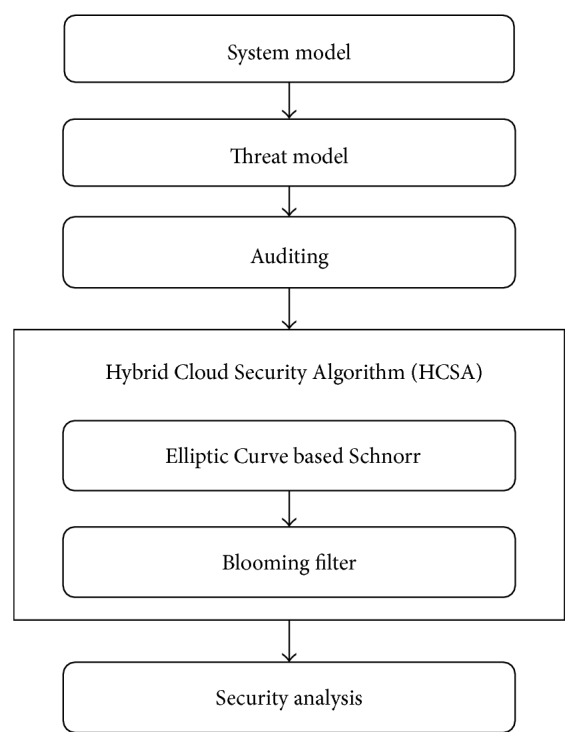
Flow diagram of proposed method.

This section presents the detailed description of the proposed Hybrid Cloud Security Algorithm (HCSA) in the cloud security model. The flow diagram of the HCSA implementation is shown in Figure 2. The workflow comprises various processes such as system model, threat model, auditing, signature set creation/verification, and duplication removal to improve the security performance. Initially, the cloud security model is created in two stages, namely, system model and threat model. Then, the auditing process is performed on the created models to address the various security issues and attacks. Then, an Elliptic Curve-Schnorr scheme based encryption/decryption performed on cloud security model and, finally, the application of the enhanced bloom filter concept to EC-Schnorr result in that enhanced the security performance with less overhead and execution time.

### 3.1. System Model

**Figure 3:**
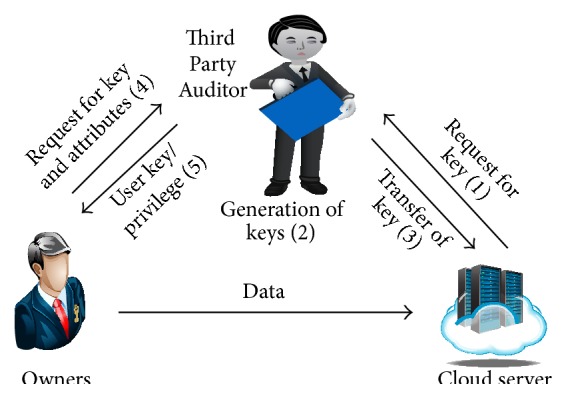
System initialization.

The cloud security model contains three modules, namely, data owners (cloud users), cloud server, and Third Party Auditors as shown in Figure 3. The cloud users store large amount of data in the cloud. Initially, the data owners computes metadata of user data without considering cryptographic keys. Cloud server is monitored by Cloud Service Provider (CSP), which provides the data storage space and computation resources. The capability of Third Party Auditor (TPA) is to improve the reliability of cloud data storage. The users dynamically interact with cloud server for accessing and updating stored data in various applications. The computation resources and burden are reduced by ensuring the integrity of outsourced data. The attacks introduced in cloud server significantly affect the integrity.

### 3.2. Threat Model

**Figure 4:**
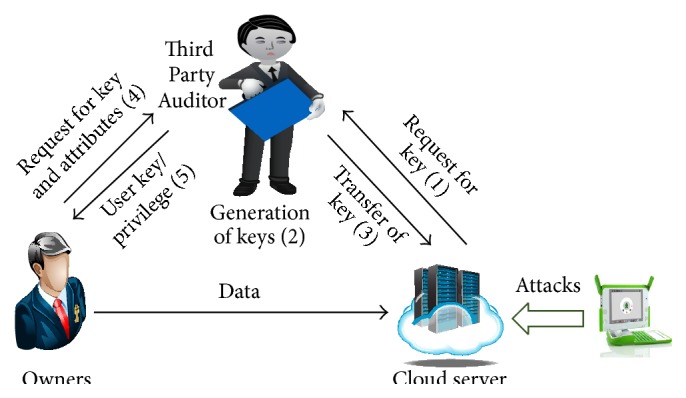
Threat model.

The system model creation is based on the consideration of the Third Party Auditor (TPA) to be genuine and mysterious. Hence, the privacy requirement for auditing protocol is necessary to create mysterious TPA. The assumption for creation of TPA is that none of the data are leaked out during the auditing process. But the attacks in threat model cause the data leakage. The threat model analyzes the attacks in server as shown in Figure 4.

The server in the cloud system model handles the three types of attacks, namely, replace, reply, and forge attacks. 

*Replace Attack.* The replacement of original metadata (*m*_*i*_, *t*_*i*_) with the uncorrupted pair of data (*m*_*k*_, *t*_*k*_) denotes the replace attack.

*Replay Attack*. The new proof generation from the existing without referring data originality introduces an attack called replay attack.

*Forge Attack*. The enabling of metadata of user data misguides the auditor leads to forge attack.

### 3.3. Auditing

The Third Party Auditor (TPA) monitors the integrity and status of outsourced data. The assumptions for auditing process are as follows:

1. TPA is reliable and independent.
2. TPA evaluates and monitors the integrity and availability of delegated data on regular intervals.
3. TPA supports the dynamic data operations.

**Figure 5:**
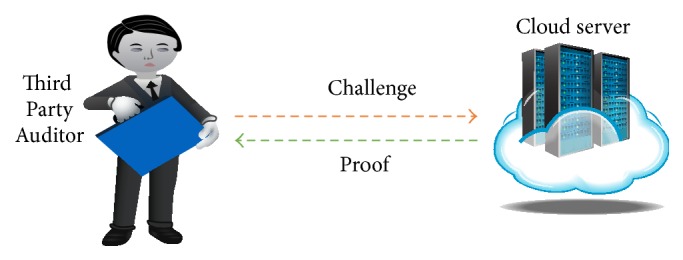
Auditing process.

Auditing process is grouped into three processes, namely, tag generation, periodic sampling audit, and dynamic operations. Initially, tag generation process groups the *n* blocks to generate the verification parameters and index hash values constituted secret key *S*_*k*_. Random sampling audit process accepts the retrieval proof in response to broadcast of challenges in random sampling as shown in Figure 5.

User application contained secret key *S*_*k*_ derived from index hash table (IHT) update and outsourced file *F* manipulations. An outsource file consists of *n* blocks of messages {*m*_1_, *m*_2_,…, *m*_*n*_} and each block of *m*_*i*_ grouped into *s* sectors like {*m*_*i*,1_, *m*_*i*,2_, *m*_*i*,3_,…, *m*_*i*,*s*_}. The tag pair of messages *m*_*i*_ contains signatures *σ*_*i*_ and secrets *S* = *τ*_1_, *τ*_2_,…, *τ*_*s*_. The convergence of *s* blocks is estimated with the help of Elliptic Curve-Schnorr algorithm.

### 3.4. Hybrid Cloud Security Algorithm

The auditing process in this paper is based on the Hybrid Cloud Security Algorithm (HCSA) and comprises two phases, namely, Elliptic Curve based Schnorr Algorithm for signature proof creation/validation and blooming filter to avoid the duplication entry. Initially, the message field and domain parameters are applied to Elliptic Curve based cloud security model to create Schnorr signature set. Then, generation and verification of proof carried were out based on Distributed Hash Table (DHT) entries. Finally, blooming filter was applied to eliminate the multiple entries in DHT. The outsourced file *F* is represented by Weierstrass's equation given as follows:

(1) $$\begin{array}{c} y^{2}=x^{3}+ax+b,\;a,b\in F. \end{array}$$

The EC- Schnorr based cryptography domain consists of various parameters listed in Table 1. The proposed HCSA for cloud auditing process is as in Algorithm 1.

The algorithm accepts public and private keys and domain parameters (*p*, *a*, *b*, *L*, *n*, *h*) for key generation process. The client in cloud security model generates public *P*_*k*_ and private keys *S*_*k*_. The base point *L* corresponding to the field *F* is chosen on elliptic curve *E*(*F*). The pseudorandom number *S*_*k*_ within the range (1 ≤ *S*_*k*_ ≤ *n*) is selected. The public key *P*_*k*_ is calculated from pseudorandom number *S*_*k*_ and base point *L* by using the following equation:

(2) $$\begin{array}{c} P_{k}=S_{k}\cdot L. \end{array}$$

Finally, the key pair (*P*_*k*_, *S*_*k*_) were generated and they were regarded as an output. Then, HCSA accepts public (*P*_*k*_) and private key *S*_*k*_, file block *F*, and selected point *L*. Random number *K* is generated within the ranges of (1 ≤ *K* ≤ *n* − 1). The hash value for message blocks is generated by using the following equation:

(3) $$\begin{array}{c} e_{i}=H\left(\;m_{i}\;\right). \end{array}$$

The new point (*x*_1_, *y*_1_) with reference to base point (*x*, *y*) and pseudorandom number (*K*) is computed by using the following equation:

(4) $$\begin{array}{c} KL=\left(\;x_{1},y_{1}\;\right). \end{array}$$

The signature set (*σ*, *τ*) is calculated by using the following equations:

(5) σ=x1,τ=K−1ei+Skσmod n.

The process of extracting the signature was iteratively done until all the messages in outsourcing field were taken out.

The algorithm generates the proof that contains a tag, Auxiliary Authentication Information (AAI), and index hash table coefficients *H*(*m*_*i*_) as a proof. The hash value is calculated by dividing the new entry by the length of the table. The remainder is the required position to insert the new item. The hash value from the distributed table is utilized to generate the proof. The HCSA verifies the generated proof with the Boolean values of TRUE and FALSE. The authenticated message, hash value, authenticated public key, and domain parameters are arranged as proof and then verify whether the generated signature is valid or not. The algorithm accepts the signature outputs (*σ*, *τ*) from SignGen. Then, the status (TRUE or FALSE) of signature set is identified by using the condition *σ* ∈ {0,…, 2^*l*^ − 1} and *τ* ∈ {1, 2,…, *n* − 1}.

The false report of validation terminates the process. Otherwise, the new point is calculated according to following equation:

(6) $$\begin{array}{c} L_{new}=\left[\;\tau \;\right]L+\left[\;\sigma \;\right]P_{k}. \end{array}$$

The process continued on the basis of *L*_new_. The termination occurs for zero values of *L*_new_ and the process continued for nonzero values of *L*_new_. Then, two processes such as Octet String to Integer (OS2I) and Finite Field Element to Octet Series (FE2OS) are involved in the verification process. The OS2I and FE2OS of hash value and public key (*P*_*k*_) are stored in the temporary variable *temp*. Finally, the comparison between *temp* and signature *σ* provides the status (TRUE or FALSE) of proof.

### 3.5. Blooming Filter

The probabilistic data structure to predict the member in a set with minimum false positive rates is referred to as blooming filter. The use of large bit array in blooming filter concept efficiently reduced the false positive probability. The bloom filter contains the positions of bit corresponding to existing entries. The logger generates the *K* number of bit positions for each entry by hashing of *n* times and updates the data entry with new Accumulator Entry (AE). The outsourced message field *F* = {*m*_1_, *m*_2_,…, *m*_*n*_} contained *n* set of entries arranged into a membership function (*M*) of bit vector *B* length *n*. The hash functions *H* = {*h*_1_, *h*_2_,…, *h*_*n*_} with *h*_*i*_: *x* → {1,…, *n*} were computed initially. Then, the filter coefficients are computed by allocation of *n* bits to zero. The algorithm for filter coefficients prediction is shown in Algorithm 2.

The testing of an element in the membership function returns the TRUE for the presence of an element and FALSE for absence of element in membership. The number of hashing functions applied to determine the status of filter in testing phase significantly reduces the storage complexity and execution time.

## 4. Performance Analysis

This section presents the performance analysis of the proposed Hybrid Cloud Security Algorithm (HCSA) regarding execution time, storage complexity, and security. The Elliptic Curve based Schnorr signature generation and blooming filter prediction enhanced the security performance compared to Distributed Hash Table (DHT).

### 4.1. Storage Complexity

The storage complexity of Cloud Service Provider (CSP) depends on outsourced data file *F* and hash value. CSP contains the outsourced data file *F* = {*m*_1_, *m*_2_,…, *m*_*n*_}, data block tags *T*, and random chosen point *K*, which are used to compute the digital signature denoted as hash value *H*(*m*_*i*_). The cost of storage *C*_CSP_ and the storage complexity *S*_CSP_ are calculated by

(7) CCSP=F+T+K+Hmi,SCSP=SCSPn.

**Figure 6:**
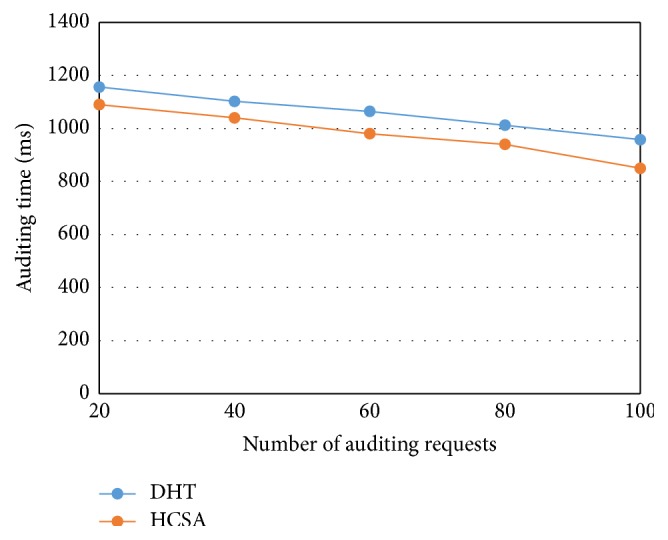
Auditing time versus number of auditing requests.

Figure 6 depicts the auditing time variation with the number of auditing requests. The total number of auditing requests in our proposed model is 100. The DHT and proposed HCSA consume 1156 and 1090 ms for minimum auditing requests handling. Also, they consume 958 and 850 ms for maximum auditing requests. The comparison shows the proposed HCSA algorithm offer 5.71 and 11.27% reduction for minimum and maximum requests compared to existing DHT due to the duplication elimination.

### 4.2. Execution Time

The execution time is the time required from challenge creation to proof verification. The execution time *t*_exec_ of TPA depends upon various parameters, namely, time for challenge creation (*t*_chall_), *K* pseudorandom permutations (*K* × *t*_PSP_), *K* pseudorandom functions (*K* × *t*_PSF_), time for proof creation (*t*_pr_), time for signature verification (*t*_very_), and comparison time of proof (*t*_comp_) in cloud server defined by

(8) $$\begin{array}{c} t_{exec}=t_{chall}+K\times t_{PSP}+K\times t_{PSF}+t_{pr}+t_{very}+t_{comp}. \end{array}$$

**Figure 7:**
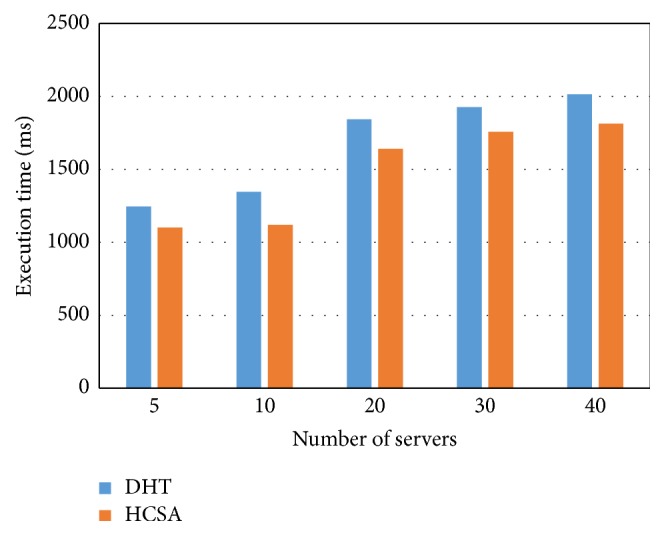
Execution time versus number of servers.

Figure 7 depicts the execution time variation with number of servers. The total number of servers in our proposed model is 40. The DHT and proposed HCSA consume 1245 and 1100 ms for minimum servers. Also, they consume 2014 and 1814 ms for maximum servers. The comparison shows the proposed HCSA algorithm offers 11.65 and 9.93% reduction for minimum and maximum requests compared to existing DHT due to the ECC-based signature creation with the optimized steps.

### 4.3. Computational Overhead

**Figure 8:**
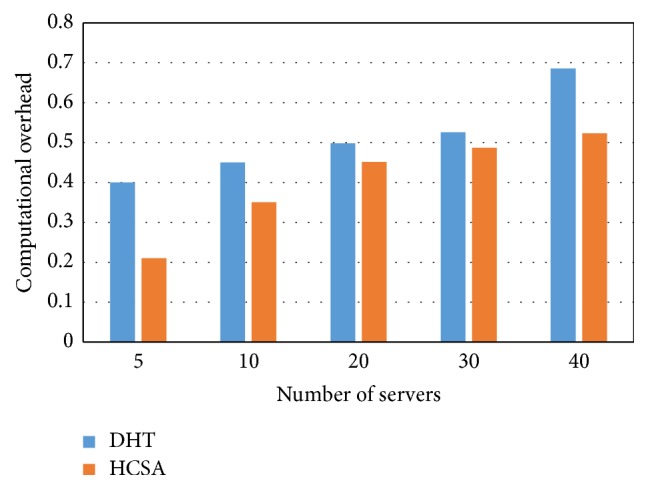
Computational overhead versus number of servers.

The computation overhead is lesser than the DHT models.  Figure 8 depicts the computation overhead with respect to the number of servers. The total number of servers used in our proposed model is 40. For each server, the computation overhead with HCSA is lower than the DHT model.

The increase in number of servers gradually increases the computational overhead generally. But the optimization and duplication removal by using the proposed algorithm provide lesser computational overhead for minimum (47.5%) and maximum servers (23.69%) compared to existing DHT.

## 5. Conclusion

The proposed Hybrid Cloud Security Algorithm (HCSA) presented the solution to the problems in secure cloud data storage system modelling based on the combination of Elliptic Curve based Schnorr (EC-Schnorr) scheme and blooming filter. The efficiency of the system improved and the storage complexity is reduced by removal of nonrelated contents and duplication. The malicious activity prediction was improved by using the proposed trust evaluation model. Moreover, blooming filter concept applied to the security model to avoid the cloud server. The optimization in the computational steps by ECC signature set and the duplication removal by blooming filter in the proposed Hybrid Cloud Security Algorithm (HCSA) effectively reduced the execution time, computational overhead, and auditing time with the number of auditing requests and servers. The comparative analysis between the HCSA-based model and Distributed Hash Table (DHT) model confirmed the effectiveness of proposed hybrid method of encryption schemes in cloud security model creation.

## Figures and Tables

**Algorithm 1:**
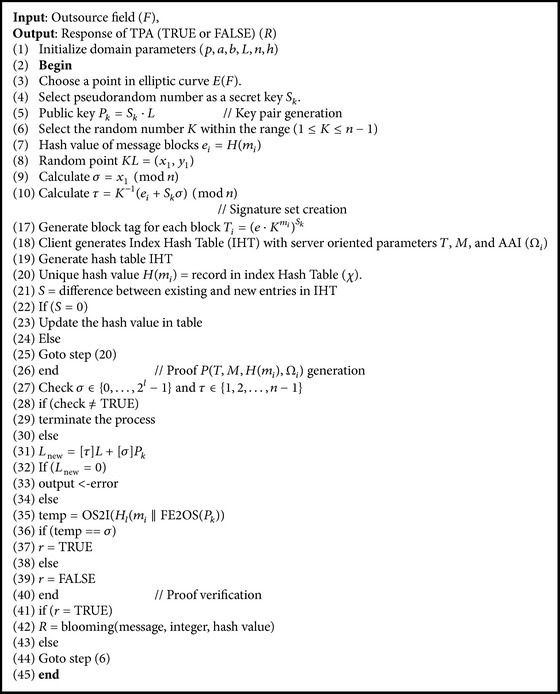
Hybrid Cloud Security Algorithm.

**Algorithm 2:**
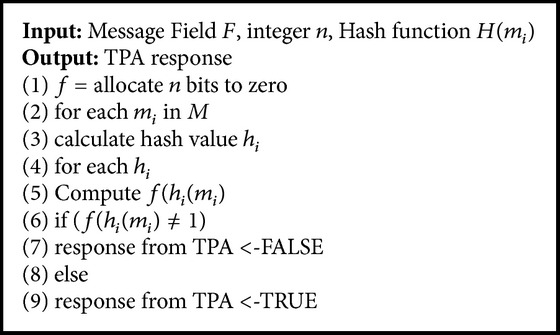
Filter coefficient prediction.

**Table 1:** Parameters.

Parameters	Description
*p*	Prime number
*a*	First coefficient in Weierstrass's equation
*b*	Second coefficient in Weierstrass's equation
*L*	Base point
*n*	Order of *L*
*h*	Cofactor of *L*
